# Supplementary material for: STAMP: Simultaneous Training and Model Pruning for low data regimes in medical image segmentation
Source: Med Image Anal. Author manuscript; Available in PMC 2026 Mar 14. (PMC7618873; doi:10.1016/j.media.2022.102583)
Supplement: Supplementary Material [file EMS212827-supplement-Supplementary_Material.pdf]

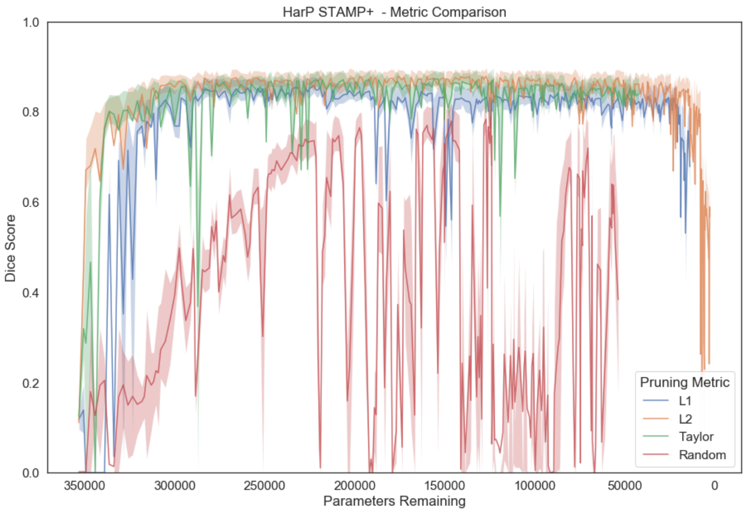

Figure 18: The performance of the *STAMP+* algorithm with different choices of pruning metric using the HarP data, with $f = 4$. It can be seen that the three metrics performed similarly, with no significant difference between L2 and Taylor metrics. All three metrics were better than random pruning. Note that the results are plotted against the remaining parameters rather than the pruning iteration.

9. Supplementary Material

9.1. Choice of Metric

Here, we explored the choice of metric used to determine which filters to prune. As stated in section 2, the L2 norm was chosen as the pruning metric and the results shown in Fig. 18 formed the basis of that decision. For N data points, the L2 norm was compared with:

- L1 norm (Li et al., 2017): $\Theta_{L_1}(\boldsymbol{z}_l^{(k)}) = \frac{1}{N}\sum_{i=1}^{N} || \boldsymbol{z}_{l,i}^{(k)} ||_1$
- Taylor – proposed in (Molchanov et al., 2016), which utilises the product of the activation and the gradient of the loss function: $\Theta_{TE}(\boldsymbol{z}_l^{(k)}) = \frac{1}{N}\sum_{i=1}^{N}(||\frac{1}{M}\sum_{m=1}^{M}\frac{\delta C}{\delta \boldsymbol{z}_{i,l,m}^{(k)}}\boldsymbol{z}_{i,l,m}^{(k)}||_1)$ where m is the element in the vectorised feature map of length M.
- Random: $\Theta_{Rand} \sim Uniform(0, 1)$

where all the metrics but Random are normalised according to Equation 3. This normalisation ensured that the last filter at any depth was not pruned. With Random pruning, the condition that the last kernel at a given depth cannot be pruned was explicitly coded. Figure 18 shows the Dice scores on the test dataset at each pruning iteration for models trained using *STAMP+* and with each metric in turn. For each metric, the mean value across the test set is shown as the solid line, and the shaded region indicates the interquartile range. The results are plotted against the remaining parameters rather than the pruning iteration. This was done because different pruning iterations remove different numbers of parameters, depending on network location from which the filter was removed: for instance, if it also led to filters being removed across the skip connection.

It can first be seen that all three metrics performed better than randomly pruning channels, as would clearly be expected. As the models started from random initialisation, the performance initially for all four metrics was poor, then improved rapidly for all metrics except random as the model training continued. It can then be seen that all three of the metrics performed comparably on this task, with no significant difference between the performance of the L2 and Taylor metrics (L2 vs Taylor: $p = 0.07$, L2 vs L1: $p = 0.003$, L2 vs Random: $p = 5.7 \times 10^{-11}$). As the L2 norm is computationally more efficient than the Taylor metric, it was used throughout the experiments.

9.2. Number of filters - Further exploration

The *STAMP+* algorithm was tested on the HarP data for models with varying numbers of filters: $f = [2, 4, 8, 16]$. Each model was simultaneously pruned and trained until the model was unable to be pruned any further, leaving only one filter remaining in each layer. Figure 19a shows the performance of the model: the performance was evaluated on the test set for each iteration of the pruned model; the mean value (the solid line) and the interquartile range bounds (shaded) are shown. The results are shown for four different initial sizes of model, plotted against the number of parameters remaining in the model. For comparison, the correspondingly sized *Standard UNet* models were also trained until convergence.

First, it can be seen that the model could be pruned and trained simultaneously, such that the same network performance was reached as the model trained to convergence; therefore, the pruned network was sufficiently powerful to be able to represent the variation in the data. It is also evident from Fig. 19 that it was possible to prune the models to a fraction of their original size without reducing the network performance. We also found that the smallest model, when $f = 2$, was not able to perform the segmentation well (maximum Dice score $= 0.788 \pm 0.052$ for the standard UNet and 0.799 ± 0.050 for the pruned model); however the larger networks could be pruned to the same number of parameters as the $f = 2$ model (and smaller) and still perform well on the segmentation task. This indicates that by pruning a larger model we were able to learn a better arrangement of parameters than when naïvely creating a model of that size.

9.3. Shallower Network

To allow further exploration of the effect of the initial size of the model, pruning a shallower original model was also considered. A downsampling and an upsampling layer were removed, so the original model was smaller, with fewer parameters, but still having the ability to learn the same number of features at the layers of abstraction that remained. A shallower network was considered, rather than a deeper one, as it allowed the exploration of another way in which the model could be smaller in terms of parameters. This architecture can be seen in Fig. 20. All other parameters were held the same as in the experiments with the full-sized model and the value of f was varied.

We first investigated results obtained using the HarP data, for different values of $f = [2, 4, 8, 16]$. Figure 21 shows the results, where it can be seen that the results with the shallower UNet followed the same pattern as was seen with the *Standard UNet* - Fig 19. Again, it is clearly visible that a better performance on the segmentation task was achieved

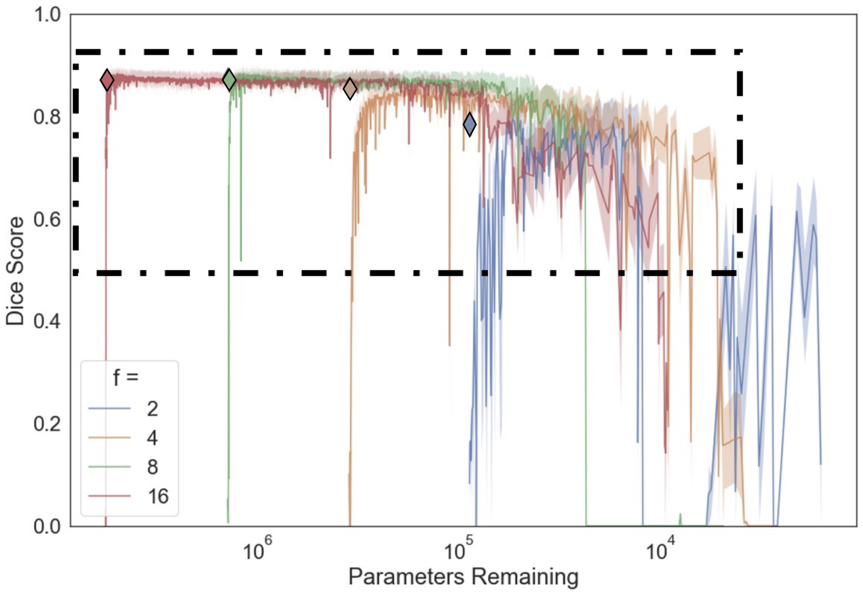

(a)

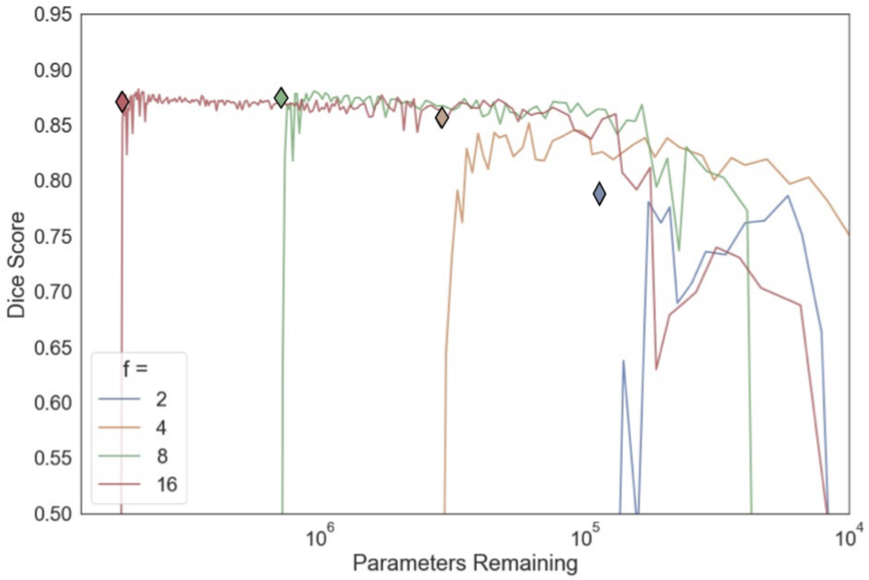

(b)

Figure 19: a) Results for pruning models of varying model sizes – using different values of f – while training to segment the hippocampus on the HarP data. The black diamond corresponds to a model of the same size being trained to convergence on the same data, with all hyper-parameters held the same except for the absence of pruning the network. b) The same result, zoomed in and subsampled (only every 10th data point shown, only the mean, with no interquartile range shown) to allow the result to be seen more clearly. The box on a) indicates the zoomed in region.

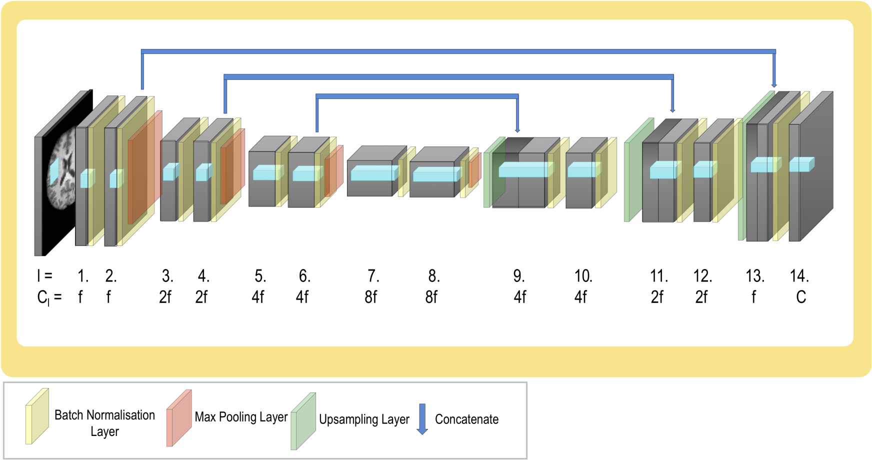

Figure 20: Shallower UNet model architecture - it follows the standard pattern of halving resolution and doubling filters at each depth but has less depth than the UNet architecture previously considered. l corresponds to the layer depth, C_l is the number of channels in that layer and C is the number of classes in the output segmentation.

if we began with a larger model and pruned it to be the same size as the smaller model, rather than originally training the smaller model to convergence. The pruning was, however, less stable, showing that the model training was less robust to filters being removed than when the model was deeper.

Fig. 22 compares the results from the shallower UNet to the original UNet when the original models were matched in terms of parameters, where Fig. 22a compares an original depth UNet with $f = 4$ to a shallower UNet with $f = 8$, and Fig. 22b compares an original depth UNet with $f = 8$ to a shallower UNet with $f = 16$. The black diamonds represent the original *Standard UNets* trained to convergence; they performed very similarly on the data. As they are pruned, the two similarly sized networks had very similar performance on the main task, indicating that the exact arrangement of the filters is not vital for the model's performance. This therefore suggests that the algorithm was reasonably robust to the initial model configuration and the results were not due to the initial choice of model architecture. This is also demonstrated by Fig. 23, which shows the distribution of filters during training for the shallower UNet when training with the HarP data. It can be seen that, as is evident in the other examples shown, the bottleneck of the model was pruned aggressively early in the pruning, and the first layer at each depth was pruned first – the result shown is for $f = 8$ but the pattern was the same for all values of f considered.

9.4. Method Comparison

In Fig. 24 the results achieved using different pruning methods are compared: *STAMP+*, *PruneFinetune*, and *STAMP*. First, it can be seen, by comparing *STAMP* with and without targeted dropout, that the targeted dropout both improved the model's performance (from 0.844 ± 0.03 to $0.879 \pm 0.02, p < 0.001$) and additionally made the training more stable. This shows that the targeted dropout successfully made the model more robust to being pruned, even with this relatively simple task.

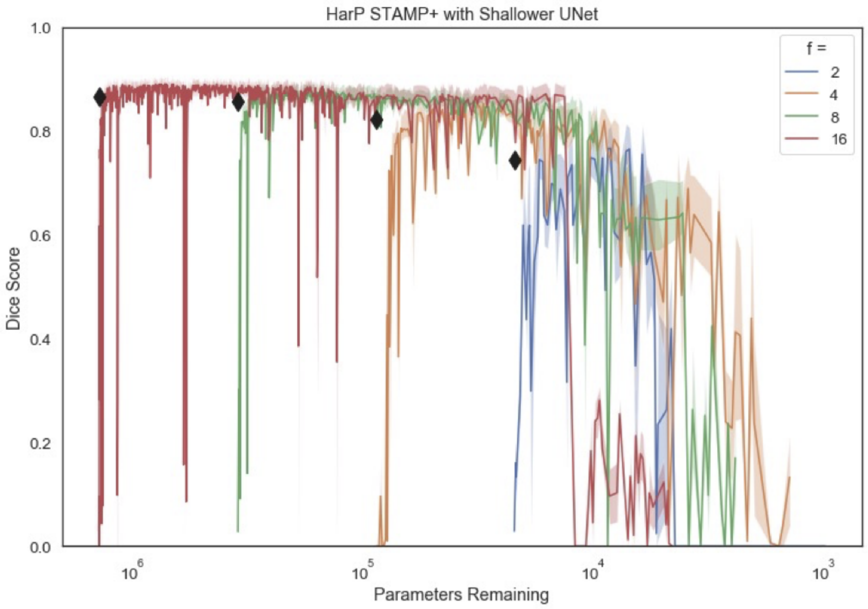

Figure 21: Dice scores on the HarP data using the shallower UNet architecture for $f = [2, 4, 8, 16]$, with interquartile bounds shown, using *STAMP+*. Diamonds represent a *Standard UNet* with that number of parameters trained to convergence.

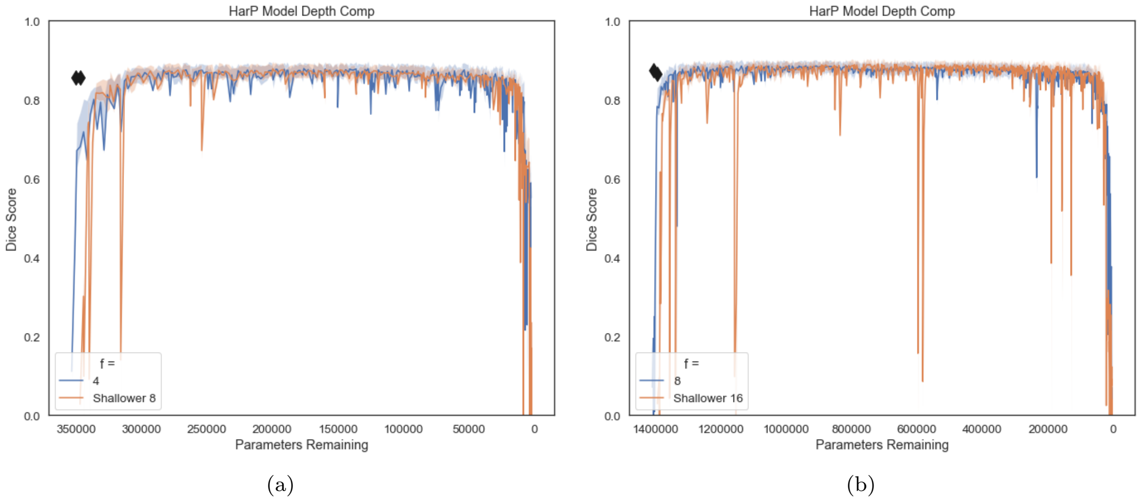

Figure 22: Direct comparison of pruning the original UNet considered – Fig. 3 – and the shallower UNet – Fig. 20 – for the case where the two models have similar numbers of parameters originally: a) compares the UNet with $f = 4$ to the shallower UNet with $f = 8$. b) compares the UNet with $f = 8$ to the shallower UNet with $f = 16$. The black diamonds represent the original *Standard UNets* trained to convergence.

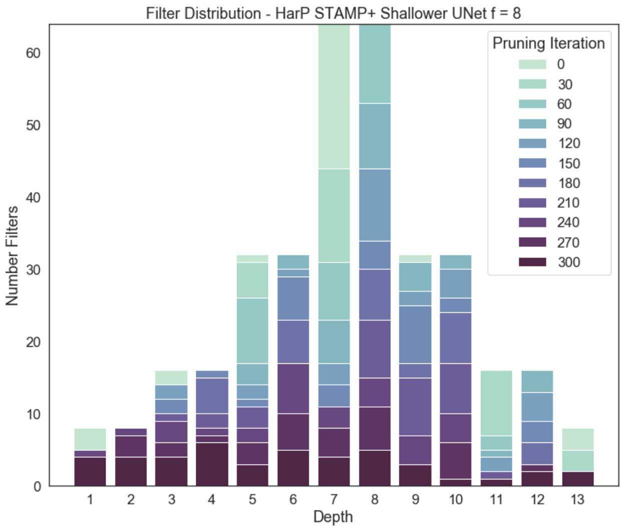

Figure 23: The distribution of the filters with network depth as the shallower model is pruned for hippocampal segmentation on the HarP data, where the darker the shade of the filter block, the longer the filters at that depth were maintained in the model.

Comparing *STAMP* to *PruneFinetune*, we can see that pruning the already converged model leads to more stable training than *STAMP* (Fig. 24). However, the removal of a single filter at a time is very conservative compared to the normal approaches taken in the literature, where a percentage of filters, or all filters under a threshold value are removed (Cun et al., 1990), which we would then expect to be less stable. Furthermore, the recovery time between filter prunings is much longer, as the model is allowed to recover entirely before additional pruning is performed (representative training graphs can be found in the supplementary material). Finally, the original had to be trained to convergence before the network could be pruned, and so *PruneFinetune* has a much larger computational cost while performing less well than the proposed method of *STAMP+* ($0.860 \pm 0.03, 0.879 \pm 0.02, p < 0.001$). Therefore, the proposed method allows better performance to be achieved without having to train the original model.

9.5. Training Graphs

Figure 25 shows representative training curves for the two approaches to pruning, with a) showing *STAMP+* during training and b) *PruneFinetune*. The region indicated in red shows the epochs used to train the initial *Standard UNet* model which was used as the initialisation for the pruning process. It can clearly be seen that *STAMP+* requires a fraction of the epochs to train and that the validation performance is much more stable throughout the process.

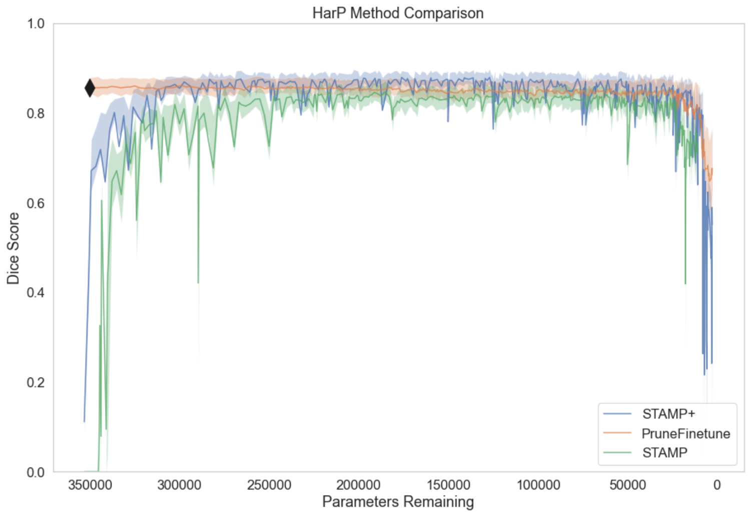

Figure 24: Comparison of pruning the HarP data with $f = 4$ for our proposed method, *STAMP+*, *Prune-Finetune* and *STAMP* (i.e. without targeted dropout - green blocks from Fig. 1 removed). The black diamond indicates the performance of the *Standard UNet* trained to convergence without any pruning – $f = 4$.

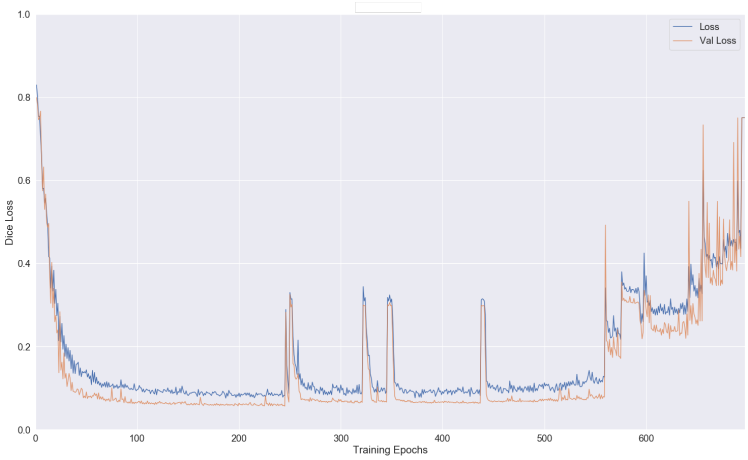

(a) STAMP+

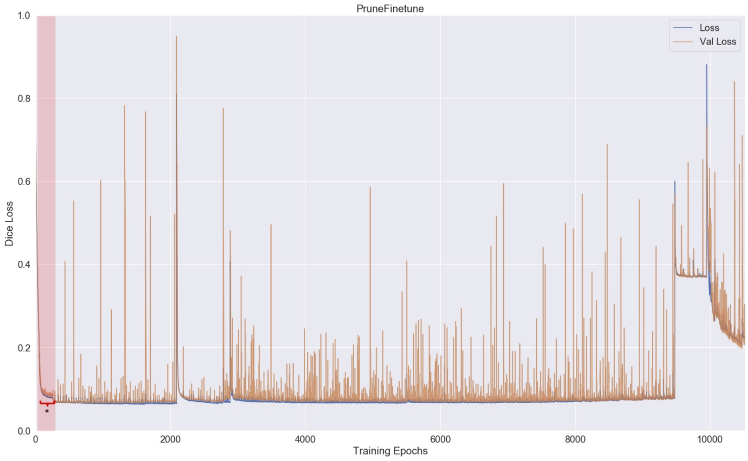

(b) *PruneFinetune*

Figure 25: Training and validation loss curves for a) *STAMP+* and b) *PruneFinetune* for the HarP data. The region indicated in red for *PruneFinetune* indicates the epochs for training the initial UNet model.

9.6. *IXI Subcortical Segmentation*

Table 2 shows the results for the IXI subcortical segmentation with increasing amounts of training data, broken down by subcortical region being segmented. It can be seen that when there is insufficient training data to train the *STAMP+* method, the failure mode is the same as the *Standard UNet*, with the network failing to segment one of the subcortical regions.

Number of Training Images	Standard UNet			STAMP+		
	Caudate	Putamen	Thalamus	Caudate	Putamen	Thalamus
50	0.28 ± 0.07	0.19 ± 0.03	0.14 ± 0.04	0.78 ± 0.04	0.75 ± 0.04	0.0 ± 0.0
100	0.54 ± 0.12	0.65 ± 0.08	0.75 ± 0.06	0.86 ± 0.02	0.89 ± 0.04	0.93 ± 0.02
150	0.46 ± 0.06	0.78 ± 0.06	0.21 ± 0.07	0.88 ± 0.02	0.90 ± 0.03	0.92 ± 0.02
200	0.032 ± 0.01	0.0 ± 0.0	0.82 ± 0.02	0.90 ± 0.02	0.91 ± 0.03	0.94 ± 0.01
250	0.90 ± 0.03	0.080 ± 0.05	0.0 ± 0.0	0.89 ± 0.02	0.92 ± 0.03	0.94 ± 0.01

Table 2: Table showing the Dice scores of the different subcortical regions with different amounts of training data for the testing data coming from the Guy's site, comparing *Standard UNet* and *STAMP+*.

9.7. *Evaluation Metric*

Dice scores were reported throughout this work. The results for comparing the *Standard UNet* and *STAMP+* were evaluated using different metrics for segmentation performance, and are shown in Fig. 26 for the hippocampal segmentation task utilising the HarP data. It can be seen that the pattern of results was consistent across the choice of evaluation metric.

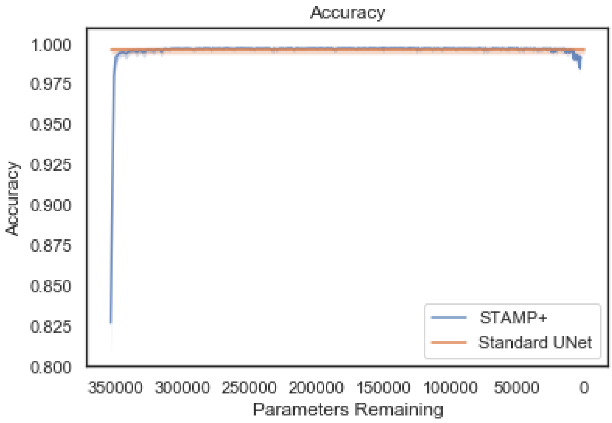

(a) Accuracy

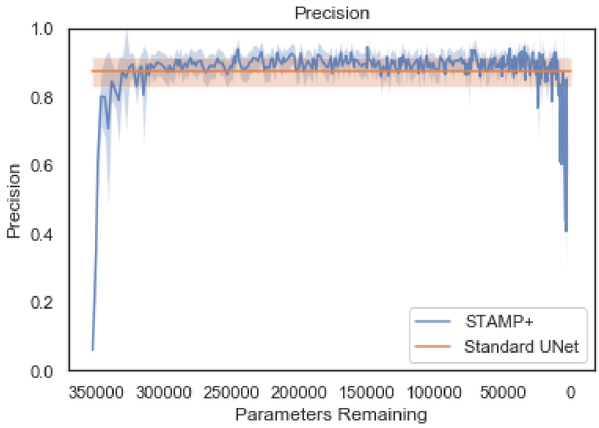

(b) Precision

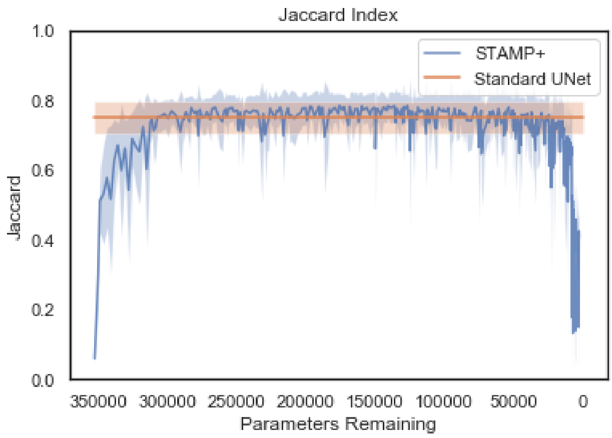

(c) Jaccard

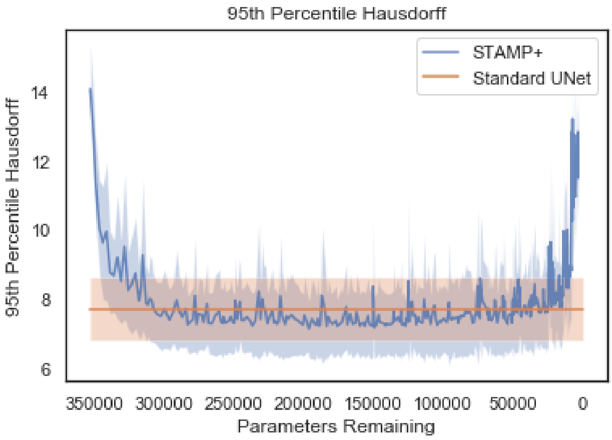

(d) 95th Percentile Hausdorff

Figure 26: Results with different evaluation metrics, comparing *Standard UNet* and *STAMP+*.

9.8. *Targeted Dropout Values*

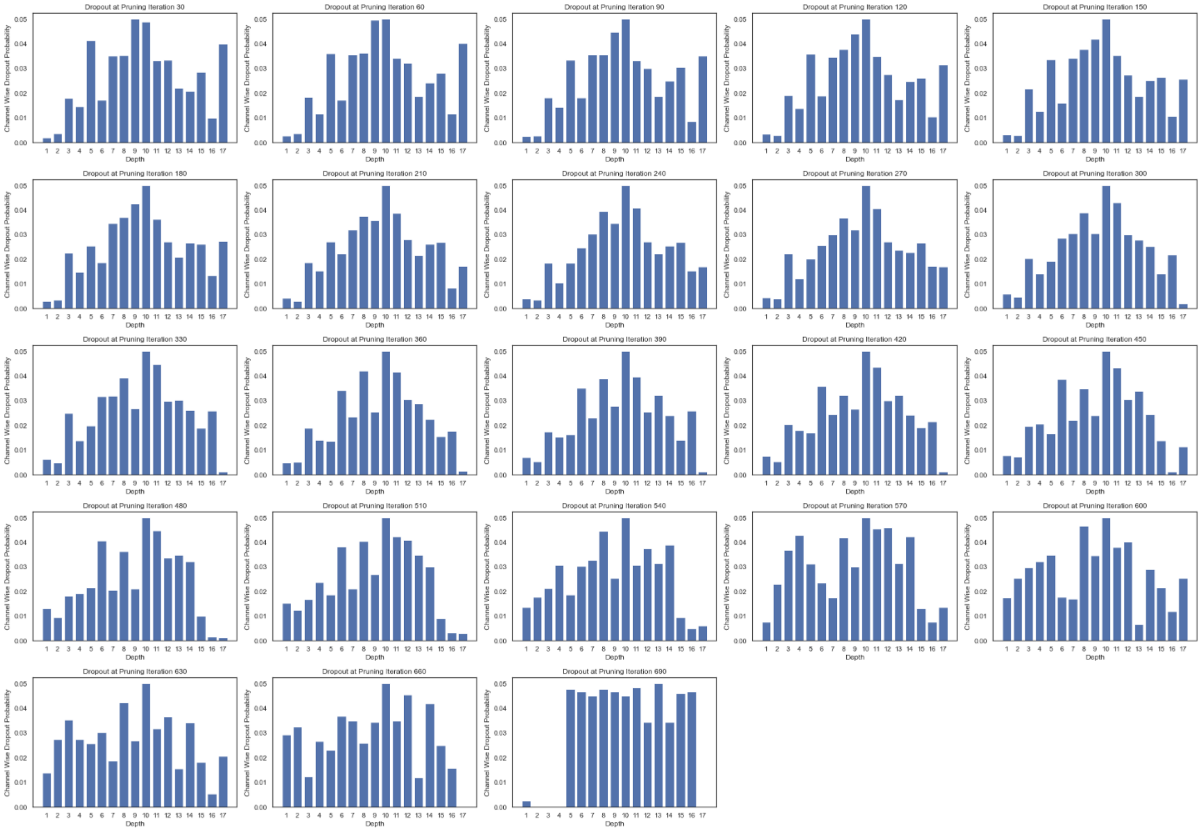

Figure 27: The adaptive targeted dropout probability values at each layer (depth) for every 30 epochs of training on the HarP data – $b_{drop} = 0.05$ – for $f = 8$. It can be seen that the dropout values are quite consistent across pruning iterations until the network becomes very small in size. When a layer has a single filter at that depth, the dropout value becomes 0, guaranteeing that information can be propagated through the model.

Remembering that the UNet architecture conventionally has doubling features with increasing model depth and halving resolution, Fig. 27 shows the adaptive targeted dropout probability values at each layer depth through training, for a model trained with $f = 8$, with every 30 iterations being reported. The x value corresponds with the location model, as indicated by the model architecture (Fig. 3). It can be seen that until the model becomes very small (highly pruned, iterations higher than 600) the distribution of the calculated values was very similar between iterations, with a tendency for the bottleneck of the model to have the highest dropout value. This was to be expected, as this is where most filters in the model are located and, thus, where there is likely to be the greatest level of redundancy. It can also be seen that towards the end of training the dropout probabilities for some of the depths are 0. These corresponded to the depth where only a single filter remains, thus to prune (or to apply dropout) would break the model and information would be unable to flow.

9.9. Filter Survival

Figure 28a shows the distribution of the number of filters at each depth as the network was pruned. The darker the section of the bar, representing the number of filters at a given layer, the longer the filters remained in the model architecture. As would be expected, as the pruning was based on the activation magnitude values, the filters in the bottleneck of the network are pruned aggressively first, corresponding to the high dropout values. It can also be seen across the network that the first layer within a pair of layers, at a given depth, is pruned more quickly than the second, for both the encoder and the decoder.

Figure 28b shows the magnitudes of the filters: $\hat{\Theta}_{L_2}(\boldsymbol{z}_l^{(k)})$ averaged across the testing dataset as the model is gradually pruned. It can be seen that as the model is gradually pruned, the average value of the remaining filters increased and the high magnitude filters remained throughout the pruning. This can be seen by considering the strong bright lines which are maintained throughout the pruning: given that a filter was initially important, it remained important throughout the training and the activations only increased in value.

The first dashed vertical line at iteration 182 represents the point in the pruning where the model had the best performance on the testing data (49% of the filters of the original model). It can be seen that the majority of the low-magnitude filters (assumed to be uninformative) have been removed, showing that more than half of the filters in the model could be removed without having a negative impact on the segmentation. The second dashed line represents the smallest network (20% of the filters of the original model) for which the performance was not significantly worse than the best performing model. This indicates that the network can be reduced to a small number of filters, where all of the activations were playing a more important role in producing the outputs.

These results clearly demonstrate that the proposed method could be utilised to simultaneously train and prune a UNet, while working in a low-data regime, as is common in medical imaging. The results also indicate the benefit of the addition of the targeted dropout, showing an increased performance, even on this relatively easy task.

9.10. Recovery epochs

We repeated the experiment presented in Section 3.4.1 on 25 and 100 data points respectively. It can be seen that the pattern is the same as that presented for 50. The more data points available for training, the lower the impact of the number of recovery epochs became.

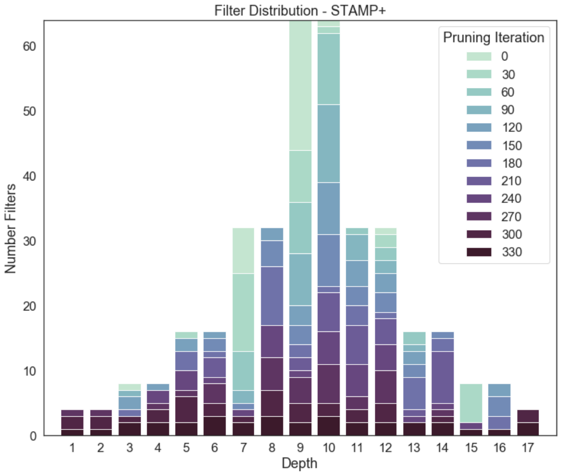

(a)

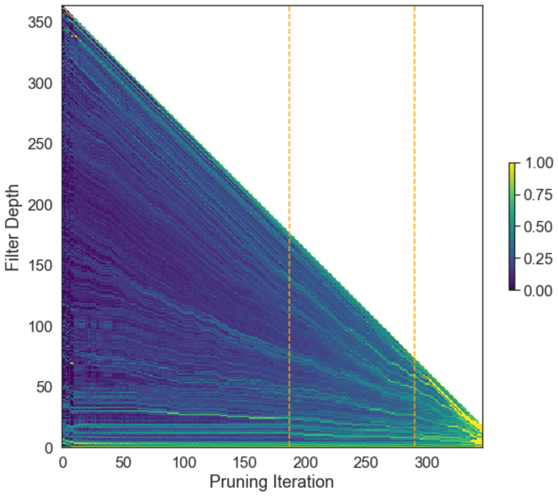

(b)

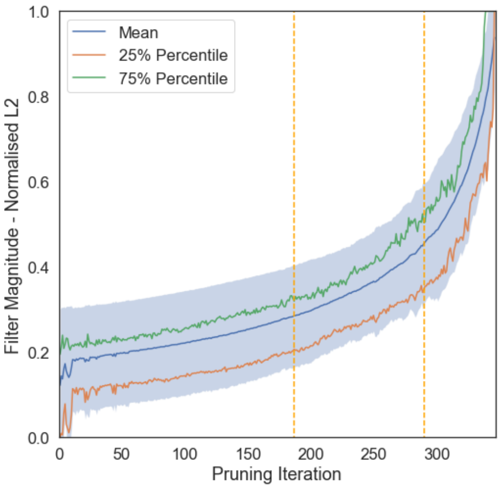

(c)

Figure 28: a) Shows the distribution of the filters with network depth as the model is pruned, meaning that the darker the shade of the filter block, the longer the filters at that depth were maintained in the models.
b) Shows the magnitudes of the activations, averaged across the training data, maintained in the model as it was pruned, where filter depth is the count of filters from the input. It can be seen that the lower magnitude activations were pruned first and the average value of the activation increased as the model was pruned. The first vertical dashed line at 182 filters corresponds to the distribution of filters which gave the best performance on the testing data. The second dashed line corresponds to the distribution of the filters for the smallest model that was able to complete the segmentation successfully, with no substantial difference in performance from the best performing model.
c) Shows the average filter magnitude, and the lower and upper quartile bounds with pruning iteration. It can be seen that the average value increased consistently with pruning iteration.

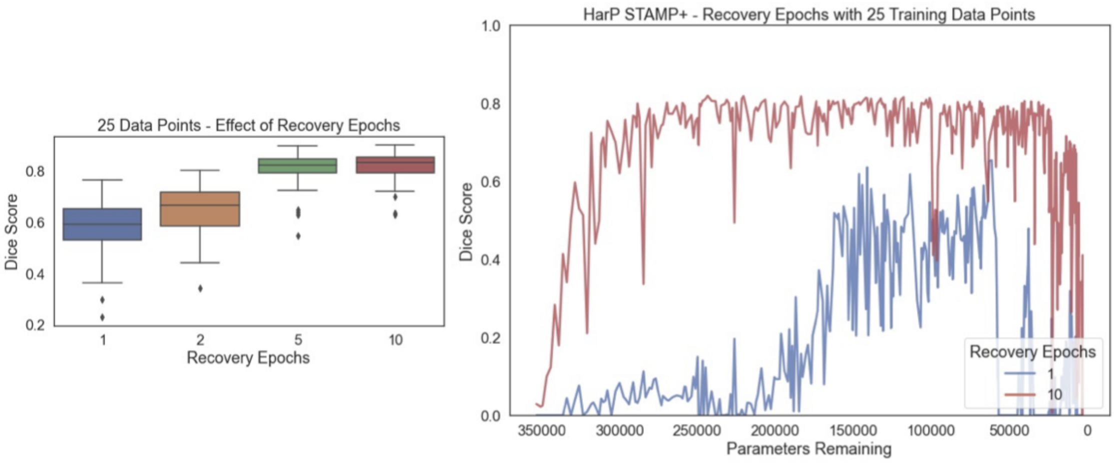

Figure 29: Effect of increasing the number of recovery epochs on the segmentation performance on the HarP data with 25 training examples. The box plot shows the best performance achieved, chosen on the validation data, and the lineplot shows the pruning dice score for 1 and 10 recovery epochs as the training progressed, showing the mean value as the number of parameters decreased.

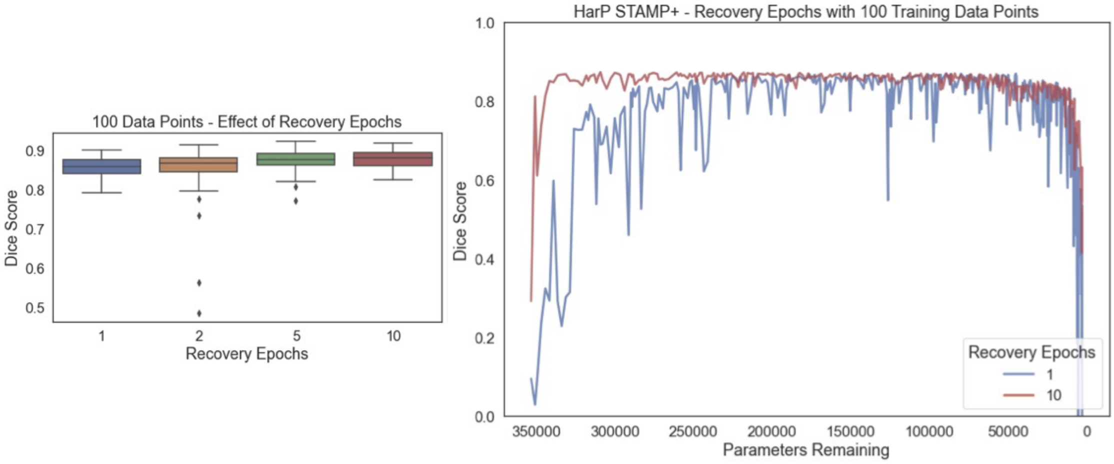

Figure 30: Effect of increasing the number of recovery epochs on the segmentation performance on the HarP data with 100 training examples. The box plot shows the best performance achieved, chosen on the validation data, and the lineplot shows the pruning dice score for 1 and 10 recovery epochs as the training progressed, showing the mean value as the number of parameters decreased.
